# Hygiene and Health Coaching for Community Readiness to Perform the Hajj during an Ongoing COVID-19 Pandemic

**DOI:** 10.3390/tropicalmed8020090

**Published:** 2023-01-28

**Authors:** Rr Suzy Indharty, Rustika, Budi Sylvana, Liliek Marhaendo Susilo, Tety Rachmawati, Zolaiha Zuchdi, Imron Cahyono, Mohammad Imran Saleh Hamdani, Asep Kusnali, Dede Anwar Musadad, Muhammad Firdaus, Al Asyary, Ziad A. Memish

**Affiliations:** 1 Department of Neurosurgery, Faculty of Medicine, Universitas Sumatera Utara, Medan 20155, Indonesia; 2 Indonesian National Research and Innovation Agency (BRIN), Bandung 40173, Indonesia; 3 Surabaya Health Laboratory Center (BBLK Surabaya), Indonesian Ministry of Health, Gubeng 60286, Indonesia; 4 Heath Center for Hajj, Indonesian Ministry of Health, Jakarta 12940, Indonesia; 5 Department of Environmental Heath, Faculty of Public Health, Universitas Indonesia, Depok 16424, Indonesia; 6 Director Research and Innovation Centre, King Saud Medical City, Ministry of Health and College of Medicine, Alfaisal University, Riyadh 11533, Saudi Arabia; 7 Hubert Department of Global Health, Rollins School of Public Health, Emory University, Atlanta, GA 30322, USA

**Keywords:** hajj, COVID-19, mass gathering, community readiness, Indonesia

## Abstract

In March 2020, WHO declared Coronavirus Disease 2019 (COVID-19) as a global pandemic, which had a major impact on all mass gatherings (MG), including the Hajj. This has an impact for the government, as the party organizing the pilgrimage can make more mature preparations for a more optimal implementation of the pilgrimage. This study aimed to evaluate hygiene and health coaching for community readiness to perform the Hajj during an ongoing COVID-19 pandemic in Indonesia. We used a mixed qualitative and quantitative method, in which the quantitative component used an analytic cross-sectional design with a questionnaire given to 2425 pilgrims, while the qualitative component was carried out through Focus Group Discussion. During the pandemic, all hygiene and health coaching, including guidance, was carried out in three types of distance learning, called “online”, “offline (face-to-face)”, and “combination”. This study shows that face-to-face health coaching is low (50.5%), while online coaching is high (70.0%). The total fraction of pilgrims who participated in blended coaching sessions was 55.1%, and the highest frequency of coaching was under four times (38.7%). However, in its implementation, there is still no integration between programs. There is quite a lot of health information given to pilgrims, but the material still varies between regions. Information on guidelines for preventing and transmitting COVID-19 for officers and pilgrims has not been fully socialized. There exists an urgent need to establish messages that are clear, meaningful, empathetic, consistent, and in context in order to achieve health improvement of pilgrims.

## 1. Introduction

**Figure 1:**
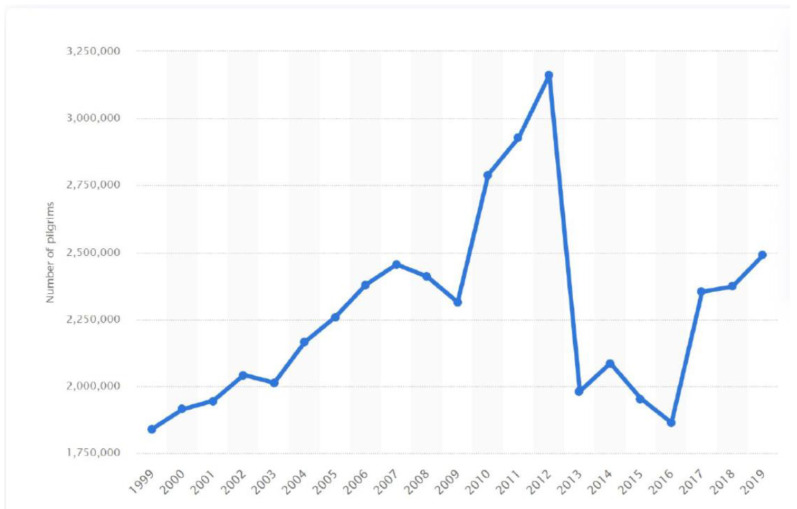
Trend in the number of pilgrims.

Mecca in the kingdom of Saudi Arabia (KSA) is a holy place for Muslims that attracts millions of Muslims from around the world every year in the month of Dhu-al-Hijjah [1,2]. Over the years, the pilgrimage has fallen in different seasons, due to the fact that the Hajj time moves back 10 days every year on the Gregorian calendar. For example, in the Hajj season of 2016, the Hajj took place in the peak summer season, with temperatures ranging between 43 °C and 48.7 °C and a relative humidity of 58–87%, thus increasing the risk of heat stress and dehydration. Performing the Hajj rites is very demanding physically, and requires pilgrims to be in exceptionally good physical condition to deal with physical stressors such as exposure to heat, dehydration, crowding, and physical activity. Moreover, due to the large number of Muslims globally and the need to save enough money to afford performing the Hajj, some pilgrims wait many years for their opportunity to perform the Hajj. Hence, a proportion of the pilgrims are over 60 years old [3,4]. The number of pilgrims performing the Hajj has increased over the years, peaking in 2012 (Figure 1) prior to the massive grand mosque expansion project and leading to an extreme increase in crowd conditions, and thus resulting in an increased risk of communicable and non-communicable diseases [4]. The average increase in the number of pilgrims per year was 5% in 2011, with the highest increase being in 2010 at around 20.6% [2].

Indonesia, with a population of around 270.20 million, is the country with the largest Muslim population in the world [4,5]. This has an impact on the number of Indonesian pilgrims who perform the Hajj. The highest increase occurred in 2017, where as many as 221,000 people performed the Hajj. Similar to what has been reported from other countries sending pilgrims, most of the Indonesian pilgrims are elderly and included in the high-risk group for having chronic diseases, and contribute to a fairly high morbidity and mortality rate each year [6]. In 2017, the percentage of at-risk pilgrims group reached 63%, while those 51–60 years old composed 35.15%, those over 61 years old 23.95%, and those over 51 years old 59.1% [4]. The most common comorbidities among Indonesian pilgrims in the last 10 years are cardiovascular diseases, respiratory infections, chronic obstructive pulmonary disease (COPD), diabetes, hypertension, and malignancies [7,8].

In March 2020, the World Health Organization (WHO) declared Coronavirus Disease 2019 (COVID-19) a global pandemic, which led the Government of KSA to halt Umra for 2020 and reduce the number of Hajj pilgrims to 1000, restricting it to locals [9]. Many other restrictions also took place in KSA, including a short period of severe lockdown with the closure of mosques and application of social preventive measures [10]. As of 9 December 2020, 67,530,912 cases of confirmed COVID-19 were reported to WHO, with 1,545,140 deaths [9,11]. Even before the announcement of the Hajj cancellation in 2020 by the government of KSA, several countries decided not to send pilgrims to KSA in fear of the further spread of COVID-19 in KSA or in their own country/globally upon the pilgrims’ return [11].

The government of Indonesia is strengthening the implementation of health istithaah (ability to perform the pilgrimage) for Hajj pilgrims, due to the large number of pilgrims who are at risk. Significant efforts are undertaken by the Indonesian Government in preparing the pilgrims every year for the Hajj following the annual requirements for Hajj released by the MoH in KSA [12,13]. The positive impact of the postponement of the Hajj pilgrimage for the Indonesian pilgrims is mainly to ensure their safety and wellbeing, in addition to giving the pilgrims more time to better prepare themselves for the next year’s Hajj [14,15].

The importance placed on sharing the right public health information with the pilgrims is critical to the success of the government’s efforts in safeguarding the pilgrims. Educating the pilgrims about hygiene, health risks, and diseases that could spread during the pilgrimage, as well as the best strategies to prevent them, is paramount. Assessment and reassessment of the knowledge gaps of pilgrims is critical in assessing the adequacy of training and communicating with pilgrims. One of the keys to dealing with public panic—such as when facing MERS-CoV, for example—is to prepare the community to face the threat by involving them in taking action in every field, including raising awareness by providing basic knowledge about the disease [16]. Based on Article 32 of Indonesian Law No. 8 of 2019 concerning the Implementation of Hajj and Umrah, health development (health conditions of Hajj pilgrim candidates) is carried out by the ministry that handles government affairs in the health sector. Coaching is carried out in an effort to improve the readiness and health status of the pilgrims, including healthy behavior and habits. This study aimed to evaluate the hygiene and health coaching for community readiness to perform the Hajj during an ongoing COVID-19 pandemic in Indonesia.

## 2. Materials and Methods

### 2.1. Study Design and Participants

This study uses a quantitative and qualitative method. The quantitative component uses an analytic cross-sectional design questionnaire given to pilgrims during the waiting period for the Hajj season of 2021, which was canceled due to the COVID-19 pandemic in Indonesia. Questionnaires were distributed through the provincial health offices.

Since COVID-19, hygiene and health Hajj coaching is carried out using distance learning. Three educational models were prepared by the Indonesian Ministry of Religion in coordination with the Indonesian Ministry of Health, namely offline, online, and a combination of the two. In offline/face-to-face coaching, the Hajj rituals involve the national public broadcasting institution media, using national television and radio broadcasting, which work together with the Indonesian Ministry of Religion and Ministry of Health. Later, prospective pilgrims are given a module containing material for the rituals of the Hajj to be studied at home. Next, they are asked to take part in the broadcast of coaching via radio and/or television to fill in the questions contained in the module. Then, on the appointed day, the results of the congregation’s homework are checked by the coaching supervisor/officer. Meanwhile, the online coaching method is conveyed through social media platforms, ranging from YouTube, to Twitter, WhatsApp, Telegram, Instagram, and Zoom. Third, a combination method (blended coaching) is applied, which integrates face-to-face/offline learning and online learning resources.

**Figure 2:**
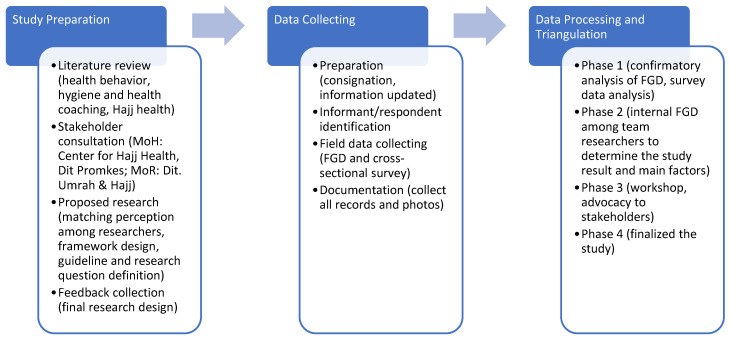
Study framework.

The qualitative component was carried out by exploring study questions that cannot be captured through the quantitative methods via Focus Group Discussions (FGD) at the level of central government, regional governments, and professional organizations. Central-government-level informants from the Indonesian Ministry of Health consist of the Directorate of Health Surveillance and Quarantine and the Hajj Health Center, while that of the Indonesian Ministry of Religion consists of the Directorate of Hajj and Umrah Guidance. Informants from each region consisted of managers of the Hajj health program at the provincial health office and the district/city health office, plus first-level and advanced health facilities that carried out Hajj health services. Informants from professional organizations consisted of the Indonesian Hajj Health Doctors Association (PERDOKHI), the Indonesian Hajj Health Association (AKHI), and the Hajj and Umrah Worship Guidance Group (KBIHU) at the research site (Figure 2).

The study was conducted in the provinces of Banten, West Java, East Java, North Sumatra, West Nusa Tenggara, South Sulawesi, and South Kalimantan. The selection of locations represents the regionalization of areas in Western, Central, and Eastern Indonesia with the highest number of congregations in each region.

### 2.2. Data Collection

Quantitative data were collected using a structured questionnaire distributed by KBIHU to pilgrims in each study location through the Google Forms application with the link https://forms.gle/kSyRKdkQmotuuuHZ9i (accessed on 20 November 2022). The Bahasa questionnaire was administered between June and July 2021. The questionnaire was adopted by Permenkes (The Ministry of Health Act) No. 15 of 2016, concerning Hajj Pilgrims Health Istithaah and Guidelines for the Prevention and Control of COVID-19. It has 4 (four) components: (1) demographic data of respondents; (2) knowledge of Hajj health and hygiene development, namely material for Hajj rituals, risk of disease in Saudi Arabia, COVID-19 disease, vaccination of pilgrims, and the role of KBIHU; (3) the attitude of the Hajj pilgrims after receiving health guidance on Hajj, namely health care, implementation of health protocols, consumption of hygiene and healthy food, medicine, and vitamins, and perceptions about dying in KSA; and (4) the behavior of the Hajj pilgrims in practicing the materials resulting from the Hajj health and hygiene coaching. The authorities (KBIHU) were ensuring that the questionnaire was filled out by each of their pilgrims. Additionally, incomplete forms were dropped with standard data cleaning before being analyzed further.

Qualitative data collection through FGDs at the central level was carried out in a hybrid manner, while at the regional level it was conducted face-to-face with strict attention to health protocols. Regional FGDs were conducted at each study location equipped with a voice recorder. The FGD guidelines refer to the variables in Permenkes (The Ministry of Health Act) No. 15 of 2016 concerning Hajj Pilgrims Health Istithaah and Guidelines for the Prevention and Control of COVID-19, which is divided into 2 (two) components: (1) health development during the waiting period, namely hygiene and health coaching and counseling; and (2) hygiene and health coaching at the time of departure consisting of hygiene and health guidance, health counseling, and integrated coaching.

### 2.3. Data Analysis

The quantitative data collected from the spreadsheet was exported into Microsoft Excel to be cleaned and coded before being processed in the Statistical Package for the Social Sciences (SPSS) software 25th version year 2021. The responses to the questions were summed and used to calculate the total score of knowledge, attitude, and behavior for each respondent. A cut-off score was given to distinguish respondents who had good knowledge, attitudes, and behavior from those who did not. The cut-off score was taken from the average number of means; if the total value was above the mean, then the value was good, whereas if the value was below the mean, then the value was bad.

Quantitative data are presented in terms of mean, standard deviation, and inter-quartile range. Certain questions (health and hygiene coaching whether conducted via the online method, face-to-face method, and/or both [online and face-to-face] methods) were analyzed using a simple frequency table. Statistical analysis was carried out descriptively with simple logistic regression (Prevalence Odds Ratio—POR) for the characteristics, level of knowledge, attitudes, and behavior of Hajj pilgrims towards Hajj health development during the COVID-19 pandemic. The target population was the number of pilgrims at the research location that planned to depart in 2022. The number of respondents was as many as 2425 pilgrims.

The qualitative data from the voice recordings of FGDs were made into a transcript and coded with the help of Microsoft Excel to obtain the thematic matrix needed based on the results of quantitative data processing. Next, the collected qualitative information was analyzed manually through conventional content analysis with an inductive approach. The policy analysis of the implementation of Hajj health development was guided by the theory of David Easton’s policy analysis system, which was further developed by Longest Beuffort (2004) in the Logic Model theory. Meanwhile, the implementation uses George Edward III’s theory of policy implementation, which explains that policy implementation is influenced by the variables of communication, resources, disposition, and bureaucratic structure. In the first step of the analysis, team members discussed the information collected internally to categorize it using interview recordings, transcripts, and interview summaries. In the second step, the entire team reviewed the results for convergence and consistency. Analysis continued throughout the writing process. Because the research instrument followed the concepts of David Easton and George Edward III, all data were analyzed with reference to those concepts.

Ethics approval was obtained from the Ethics Commission of the Health Research and Development Agency, Ministry of Health of the Republic of Indonesia in April 2021 (No. LB/02.01/2/KE.404/2021). Respondents’ data are kept confidential, and only those who gave written consent were included in the study.

## 3. Results

### 3.1. Knowledge Level of Hajj Congregants’ Attitudes and Behaviors towards Health Development

#### 3.1.1. Characteristics of Respondents

This analysis involved 2425 pilgrims. Table 1 shows the demographic characteristics of the majority of pilgrims: 65.0% age group 40–60 years, 57% female, 77% have high education, and 84.2% work. Most (98.5%) know about COVID-19, know about health coaching (64%), of that number 79% get material via YouTube, 79% have received coaching, 61.1% know the risk of disease in KSA, and 89.6% have high risk. Health coaching attitudes are generally fair (89.6%). In general, Hajj pilgrims do fitness activities (98.8%). Face-to-face health coaching is low (50.5%), while online coaching is high (70.0%). The total number of pilgrims who participated in blended coaching sessions was 55.1%, while the highest frequency of previous coaching was fewer than four times (38.7%).

#### 3.1.2. Relationship Characteristics, Knowledge, Attitudes, and Behavior Coaching

Based on Table 2 and Table 3 dependent variables are described, namely blended coaching, face-to-face coaching, and online coaching. Variables related to total mentoring were occupation, knowledge of COVID-19, knowledge of hygiene and health coaching, ability to access coaching materials via YouTube, having previously taken health coaching, knowing the risk of disease in the KSA, hygiene and healthy lifestyle attitude, fitness, and frequency of coaching (*p* < 0.05), while the variables for gender, level of education, and work are not significant (*p* > 0.05).

Face-to-face coaching shows a significant correlation with several factors, including gender, education, knowledge of hygiene and health coaching, having previously taken health coaching, knowing the risk of disease in the KSA, and frequency of previous coaching (*p* < 0.05), while the variables for age, occupation, knowledge of COVID-19, ability to access coaching materials via YouTube, hygiene and healthy lifestyle attitude, and fitness activity are not significant (*p* > 0.05).

Online coaching is not correlated with age, gender, and frequency of coaching (*p* > 0.05), while it is significantly correlated with education, employment status, knowledge of COVID-19, knowledge of hygiene and health coaching, ability to access coaching materials via YouTube, having previously taken health coaching, knowing the risk of disease in the KSA, hygiene and healthy lifestyle attitude, and fitness activity (*p* < 0.05).

In Table 3, it can be seen that face-to-face coaching is only correlated with education; highly educated pilgrims have a ratio of 1.9 times having this type of coaching compared to low-educated pilgrims (95% CI 1455 to 2573); knowledge of hygiene and health coaching status has a ratio of 1.5 times (95% CI 1.308 to 1.897); and the having undergone previous coaching four to eight times has a ratio of 1.8 times (95% CI 1.498 to 2.317). In online coaching, the occupational status has an almost 1.5 times ratio (95% CI 1.169 to 1.885). Hygiene and health coaching knowledge provides a 2.6 times ratio (95% CI 2223 to 3244). Hygiene and healthy lifestyle attitude has a ratio of 1.8 times (95% CI 1403 to 2434). In blended coaching, knowledge of hygiene and health coaching provides a 2 times ratio (95% CI 1.731 to 2.424). Hygiene and healthy lifestyle attitude has a 1.5 times ratio (95% CI 1.178 to 1.989). Those who have previously taken coaching four to eight times have a 1.5 times ratio (95% CI 1.279 to 1.889).

### 3.2. Implementation of Hygiene and Health Coaching for Indonesian Hajj Pilgrims during the COVID-19 Period

During the COVID-19 pandemic, consultation and health monitoring of Hajj pilgrims was carried out online. Pilgrims could take advantage of telemedicine services, but could not access congregation data. Hygiene and health coaching was carried out in collaboration between existing programs at the health department. In addition, collaboration between programs and KBIHU was carried out. Consultation and health monitoring was conducted online. Hajj and health coaching for Hajj pilgrims was also carried out through the COVID-19 vaccination activity for hygiene and health coaching.

*“….the use of telemedicine helps support the policies of Implementation of Restrictions on Social Activities including health services in health care facilities…. Pilgrims and their families who are able to use telemedicine have a benefit from these health services…. however, the results of examinations of pilgrims using telemedicine cannot be accessed by hajj health workers, making it difficult to monitor the health developments of pilgrims during the waiting period in the pandemic.…”*  

(Informant from Health Office in Bandung City, West Java).

*“….the health office and the Puskesmas in collaboration with KBIHU have involved TKHI candidates for health education which we conduct through webinars, some at the city level, some at the puskesmas level with TKHI, for fitness measurement we socialize it with limited face-to-face meetings conducted by several people (maximum 20 people) by implementing strict health protocols, and we do it together with prospective TKHI whose results will be inputted to Siskohatkes….”* 

(Informant from Health Office in Tangerang Selatan City, Banten).

During the COVID-19 pandemic, fitness measurements were mostly conducted independently. In 2020–2021, there was only one meeting conducted by the health office, which was carried out with limited face-to-face meetings and health protocols. Thus, the fitness measurements were carried out via self-assessment by the pilgrims themselves with family assistance, and reported using the fitness measurement form made by the Hajj Health Center. Fitness measurements were also carried out using the SIPGAR Android application developed by the Ministry of Health Kesjaor. If the results of the self-assessment were not good, then the Hajj pilgrims could consult or seek counsel from the examining doctor. Utilization of UKBM (Usaha Kesehatan Berbasis Masyarakat—Community-based Health Effort) related to health checks is intended for all people in the UKBM working area, including Hajj pilgrims.

*“….hygiene and health coaching during a pandemic is carried out online, both in the form of workshops, fitness measurements and evaluations…. during the COVID-19 pandemic physical fitness measurements were still carried out by pilgrims using the Rockport method, walking for 6 min using the Fitness Information System (SIPGAR) application.... the results obtained from 5 batches were 35,000 pilgrims in 34 provinces. There are around 26,000 pilgrims with very good results, 3612 with good conditions, 8670 enough, 8196 lacking, 8410 very lacking, 3619 pilgrims not fit….”* 

(Informant from Directorate of Occupational Health and Sport, Ministry of Health).

*“….it’s rather difficult to use the SIPGAR application because there are so many things that have to be filled in and then you have to use an email while not all congregations have email and the Android application also doesn’t necessarily work because they have to log in, so it’s really not very effective….”* 

(Informant from Health Office in Tangerang Selatan City, Banten).

During the COVID-19 pandemic, UKBM services functioned on a limited basis. The use of UKBM in relation to health checks was intended for all communities in the UKBM working area, including Hajj pilgrims. Home visits were limited to only Christian congregations with PPKM rules. Health counseling for Hajj pilgrims was carried out online through social media, such as a WhatsApp group, with the assistance of Puskesmas doctors. Dissemination of information regarding the health information of Hajj pilgrims was carried out through the WhatsApp group. Information was provided in the form of content via vlogs and flyers from the health department and the Hajj health center. Counseling with the examining doctor was conducted through limited face-to-face meetings.

*“….utilization of UKBM is usually carried out under normal conditions, health coaching is carried out by utilizing Posbindu 4 times a year, but during a pandemic it cannot be used because there is a ban on gatherings….. Posbindu is only used by the PTM program, as well as home visits during a pandemic cannot be carried out regarding pandemic policies….”* 

(Informant from Health Office in Gresik City, East Java).

*“….the counseling during this pandemic is via WhatsApp or Vlog, so we often send videos from Youtube for those counseling via the group….”* 

(Informant from Public Health Center in Gresik City, East Java).

*“….Yes, each Puskesmas has its WA group, so any information that comes to us, be it in the form of a poster or in the form of a video vlog, we will immediately forward it to the pilgrim group….”* 

(Informant from Health Office in Medan City, North Sumatera).

Several times, Hajj health counseling was conducted online together with Hajj rituals organized by the KBIHU, the Health Service, or Puskesmas as resource persons. The material was delivered in the form of pointers, vlogs, and leaflets. The healthy Hajj application has been socialized to Hajj pilgrims. Hajj health workers facilitate health counseling via telephone or smartphone using social media in sending Hajj health coaching materials. However, it is felt that the dissemination of information through mass media still does not meet the information needs of pilgrims who continue to have a high degree of technological stuttering, so the need for physical information media such as manuals, brochures, and leaflets as guides for pilgrims still exists. Usually after the health coaching, the congregation will forget about the material that has been given, and thus they still have to be provided with provisions/reading materials to read again at home. The use of mass media during the COVID-19 pandemic was carried out by forming a WA group and using YouTube to spread vlogs.

*“….in the process of health coaching and counseling during a pandemic, the Puskesmas carried out health rituals starting from health checks, supporting examinations and referrals, but the implementation had not been integrated across programs because the budgeting of health for Hajj program was not centered in one activity in the surveillance section…. KBIHU cooperates with the Puskesmas in carrying out Hajj rituals…. During the pandemic, the Hajj rituals were also carried out online and materials for the Hajj rituals were sent via e-mail or social media to the pilgrims….”* 

(Informant from Health Office in the Lombok Timur District, West Nusa Tenggara).

*“….for health education, there was quite a lot of health information given to pilgrims, but the material presented varied from one region to another…. information on guidelines for the prevention and transmission of COVID-19 for officers and pilgrims has not been fully disseminated…. health information media use more social media such as WhatsApp and YouTube in the form of limited short videos or vlogs….”* 

(Informant from Health Office in the Gowa District, South Sulawesi).

## 4. Discussion

During the pandemic, the Indonesian Government admitted that Hajj services in the era of the COVID-19 pandemic could always be improved and evaluated to be able to provide excellent service [17]. Training on excellent service in collaboration with the Religious Affairs Office (KUA) was conducted in order to improve services. There is a service satisfaction survey to assess the extent to which the government services are accepted by service users, which is one of the reasons why the congregation has a positive response regarding Hajj coaching [18].

Hygiene and health coaching was generally provided to those the prospective pilgrim candidates (CJH). CJH who received this guidance were pilgrims who had paid off and were scheduled to depart in 2020. However, due to the impact of the COVID-19 pandemic, the departure of all CJH was delayed. Although there are no guarantees regarding the next pilgrimage departure, the Hajj health team of each Health Center, together with the KUA, carried on providing health guidance. The Indonesian rule for hygiene and health guidance was carried out, including checking on the meningitis vaccination as well as the booster vaccine, which is one of the requirements of the government of Indonesia and the KSA MoH [19]. Moreover, this guideline issued by the Secretary General of the Indonesian Ministry of Health also comprised two important things for a successful Hajj in an ongoing COVID-19 pandemic. These included, first, carrying out examinations, providing hygiene and health guidance for the Hajj, and providing vaccinations in accordance with government policies. This was carried out while waiting for the certainty of the implementation of the pilgrimage. Second was recording/inputting the implementation of inspection activities, Hajj health development, and vaccination into the Health Sector Integrated Hajj Computerized System (SISKOHATKES) application [20].

According to this study’s result, face-to-face hygiene and health coaching conducted reminds CJH to seek vaccinations in accordance with government policies and statutory provisions. In accordance with the conditions of uncertainty in the implementation of the pilgrimage due to the COVID-19 pandemic, CJH must maintain their health condition so that they is ready to depart if the pilgrimage is held again. To prepare CJH health istithaah and prevent health decline during this pandemic, each region’s officers were presented to convey important information about policies for organizing the Hajj and field policies [21].

Even though this study cannot directly compare these three types of hygiene and health coaching, the evidence shows that the online learning is less effective than face-to-face learning [19]. Another study showed that direct health education and education via mass media were significantly related to hypertension treatment in adults [22]. Health education through print media is a dominant factor in treating hypertension. One study recommended regular health education through leaflets, magazines, and posters [23]. Additionally, other results stated that the online learning model can increase student activity [24]. Thus, it can be concluded that learning through online media in adults is more effective, but it may be less effective for elementary-school-age children. According to [25,26], one’s level of formal education can beconsidered the basic capital for one’s understanding of conveyed information. The higher a person’s level of formal education, the more easily it is expected that one will accept and understand the information one receives. People with a high level of education will more easily understand what they are receiving than people with less education.

In the Indonesian government, the role of main organizer of the Hajj is occupied by the Ministry of Religion, while the Ministry of Health (Center for Hajj Health) supports this agenda. The formation of regulations spread across various ministries or government agencies and local governments aims to bind government programs with the community [27]. One of them is the regulation on the implementation of the pilgrimage, in which there is a hygiene and health coach for the pilgrims. The important role of mass communication in the midst of the COVID-19 pandemic is as a form of prevention and education to the public [28]. Communication media has a central role in disseminating information. The problems caused by the COVID-19 pandemic have become global problems that have the potential to trigger a new social order or reconstruction, and thus it is necessary to have close communication between stakeholders and the community [29], including in disseminating information about hygiene and health coaching to Hajj pilgrims. In Australian pilgrims, these kinds of pre-travel health advice appeared to be obtained from Hajj travel agents rather than from medical/health professionals [30]. Additionally, practicing simple health hygiene such as the use of a facemask [31] and hand hygiene behavior may depend on each Hajj pilgrim’s knowledge [32].

The government, in communicating information related to the implementation of the Hajj and Umrah during the pandemic, must produce messages that are clear, meaningful, of one narrative, empathetic, consistent, and supported by context. In addition, there must be harmonization of communication through the integrated system between government institutions, along with guidance related to hygiene and health protocols and emotional support by Hajj officers and scholars to prospective pilgrims who fail to go to the Hajj [30]. This is important because health improvement during the pandemic is an imperative to be carried out by pilgrims.

## 5. Study Limitations

Although this research is the first study that evaluates government measures in preparing the community’s readiness to perform the Hajj during the COVID-19 pandemic, this study has limitations. In a quantitative approach, the self-assessment responses of CJH may be biased. The selected study’s locations may be also differ with other areas of Indonesian, considering the huge size of the country. However, validity was assessed in order to prevent this, with KBIHU monitoring each CJH. Additionally, the selection of respondents was administered according to CJH representativeness in each area in Indonesia.

## 6. Conclusions

During the pandemic, hygiene and health coaching methods are carried out in three types of distance learning, called online, offline (face-to-face), and combination. However, there are still some that have not been implemented in an integrated manner between programs, and not all regions can carry out online coaching activities. Health consultation and laboratory results are carried out using online services, including the use of telemedicine by pilgrims. However, the health data of Hajj pilgrims via telemedicine cannot be accessed. Physical fitness training activities are conducted in various ways, which are carried out independently and accompanied by assistance. These mentoring activities can be conducted through the WhatsApp application or via Zoom meeting, and in the field in groups with strict health protocols. The health information material for Hajj pilgrims has not been standardized, so the health information provided in each region varies. Information on guidelines for preventing and transmitting COVID-19 for officers and pilgrims has not been fully socialized. Health information media uses social media, such as WhatsApp and YouTube, in the form of short videos or limited vlogs.

Health information for Hajj pilgrims must be standardized, so that every Hajj pilgrim receives the same standardized information. It is imperative that health information reaches the pilgrims through more massive and planned outreach. Health education can be carried out using contemporary methods combined with classical methods to reach congregations who are still using these methods. It is necessary to increase the promotive and preventive health programs for the Hajj pilgrims through health coaching, to ensure that the Hajj pilgrims can independently maintain their health during the COVID-19 pandemic.

## Figures and Tables

**Table 1:** Distribution of Respondents by Characteristics.

Variable (N = 2425)		N	%
Province	Banten	129	5.3
	West Java and Central Java	616	25.4
	East Java	999	41.2
	South Kalimantan	100	41.2
	West Nusa Tenggara	147	6.1
	South Sulawesi	178	7.3
	North Sumatera	256	10.6
Age	<40 years	218	9.0
	40–60 years	1576	65.0
	>60 years	631	26.0
Sex	Male	1043	43.0
	Female	1382	57.0
Education	High	1867	77.0
	Moderate	170	7.0
	Low	388	16.0
Occupation	Works	2042	84.2
	Does not work	383	15.8
COVID-19 Knowledge	Fair	2389	98.5
	Poor	36	1.5
Hygiene and Health Coaching Knowledge	Fair	1553	64.0
	Poor	872	36.0
Able to Access Coaching Material via YouTube	Yes	1151	47.5
	No	1274	52.5
Have Previously Taken Health Coaching	Already	1914	78.9
	Not Yet	511	21.1
Know Disease Risk in the KSA	Yes	1483	61.1
	No	942	38.8
Health Risk	High	2173	89.6
	Low	252	10.4
Do Fitness Activity	Yes	2395	98.8
	No	30	1.2
Face-to-face Coaching Session	Yes	1200	49.5
	No	1225	50.5
Online Coaching Session	Yes	1699	70.0
	No	726	30.0
Blended Coaching Sessions	Yes	1337	55.1
	No	1088	44.9
Previous Coaching Frequency	>8 times	751	30.9
	4–8 times	737	30.4
	<4 times	937	38.7

**Table 2:** Proportion of face-to-face coaching, online coaching, and total coaching according to several variables.

Variable		Face-to-Face Coaching (%)			Online Coaching (%)			Blended Coaching (%)		
		Yes	No	*p*-Value	Yes	No	*p*-Value	Yes	No	*p*-Value
Age Group	<40 years	81.9	18.1	0.01	69.0	31.0	0.87	56.0	44.0	0.19
	40–60 years	73.4	26.6	0.95	78.4	21.6	0.00	56.7	43.3	0.01
	>60 years	73.6	26.4		69.7	30.3		50.8	49.2	
Sex	Male	76.9	23.1	0.01	74.1	25.1	0.70	57.0	43.0	0.11
	Female	72.3	27.7		75.6	24.4		53.7	46.3	
Education	High	77.2	28.2	0.00	77.1	22.9	0.01	54.6	45.4	0.14
	Moderate	80.8	19.2	0.01	65.9	34.1	0.00	52.1	47.9	0.57
	Low	83.2	16.8		70.5	29.5		58.8	41.2	
Occupation	Works	74.3	25.7	0.85	76.5	23.5	0.00	56.0	44.0	0.04
	Doesn’t work	73.9	26.1		68.7	31.3		50.4	49.6	
COVID-19 Knowledge	Fair	74.3	25.7	0.56	75.7	24.3	0.00	55.5	44.5	0.01
	Poor	69.4	30.6		44.4	55.6		33.3	66.7	
Hygiene and Health Coaching Knowledge	Fair	77.5	22.5	0.00	82.1	17.9	0.00	61.5	38.5	0.00
	Poor	68.6	31.4		63.1	36.9		43.8	56.2	
Able to Access Coaching Theory via YouTube	Yes	73.1	26.9	0.21	88.7	11.3	0.00	64.1	35.9	0.00
	No	75.4	24.6		63.1	36.9		47.0	53.0	
Have Previously Taken Health Coaching	Already	77.4	22.6	0.00	77.2	22.8	0.00	57.8	42.2	0.00
	Not Yet	62.4	37.6		68.1	31.9		45.0	55.0	
Know Disease Risk in the KSA	Yes	76.1	23.9	0.01	80.9	19.1	0.00	60.1	39.9	0.00
	No	71.3	28.7		66.3	33.7		47.3	52.7	
Hygiene and Healthy Lifestyle Attitude	Fair	74.1	25.9	0.70	76.6	23.4	0..00	56.2	43.8	0.00
	Poor	75.4	24.6		63.9	36.1		45.6	54.4	
Do Fitness Activity	Yes	74.4	25.6	0.21	75.6	24.4	0.00	55.4	44.6	0.03
	No	63.3	36.7		46.7	53.3		33.3	66.7	
Previously-taken Coaching Frequency	>8 times	82.8	17.2	0.00	72.2	27.8	0.12	59.8	40.2	0.00
	4–8 times	77.5	22.5	0.00	78.0	22.0	0.25	59.2	40.8	0.00
	<4 times	64.9	35.1		75.6	24.4		48.2	51.8	

**Table 3:** Odds Ratios for Hygiene and Health Coaching of Hajj Pilgrims.

Variable		Face-to-Face Coaching			Online Coaching			Blended Coaching		
		OR	(95% CI)		OR	(95% CI)		OR	(95% CI)	
			Lower	Upper		Lower	Upper		Lower	Upper
Age	<40 years	0.62	0.42	0.90	1.04	0.75	1.44	0.81	0.60	1.10
	40–60 years	1.01	0.82	1.24	0.63	0.52	0.78	0.79	0.65	0.95
	>60 years									
Sex	Male	1.27 *	1.06	1.53	0.96	0.80	1.16	1.14	0.97	1.35
	Female									
Education	High	1.94 *	1.46	2.57	0.71	0.56	0.91	1.19	0.95	1.48
	Moderate	1.65	1.11	2.46	0.57	0.41	0.80	0.90	0.66	1.24
	Low									
Occupation	Works	1.02	0.80	1.31	1.49 *	1.17	1.89	1.25 *	1.01	1.56
	Doesn’t work									
COVID-19 Knowledge	Fair	0.78	0.38	1.60	0.26	0.13	0.50	0.40	0.20	0.81
	Poor									
Hygiene and Health Coaching Knowledge	Fair	1.58 *	1.31	1.90	2.69 *	2.22	3.24	2.05	1.73	2.42
	Poor									
Able to Access Coaching Materials via YouTube	Yes	1.13	0.94	1.35	0.22	0.18	0.27	0.50	0.42	0.59
	No									
Have Previously Taken Health Coaching	Already	0.48	0.39	0.60	0.63	0.51	0.78	0.60	0.49	0.73
	Not Yet									
Know Disease Risk in the KSA	Yes	0.78	0.65	0.94	0.47	0.39	0.56	0.60	0.51	0.70
	No									
Hygiene and Healthy Lifestyle Attitude	Fair	0.94	0.69	1.27	1.85 *	1.40	2.43	1.53 *	1.18	1.99
	Poor									
Do Fitness Activities	Yes	0.59	0.28	1.26	0.28	0.14	0.58	0.40	0.189	0.86
	No									
Previous Coaching Frequency	>8 times	0.38	0.30	0.48	1.19	0.96	1.48	0.63	0.52	0.76
	4–8 times	1.86 *	1.50	2.32	1.15	0.91	1.44	1.55 *	1.28	1.89
	<4 times									

Note: * *p*-value < 0.05.

## Data Availability

All the datasets generated and/or analyzed during this study are not publicly available due to confidentiality, but are available on reasonable request.
